# Effects of tibialis anterior contraction on the medial longitudinal arch and hallux valgus angle: A functional anatomical perspective

**DOI:** 10.1371/journal.pone.0350830

**Published:** 2026-06-12

**Authors:** Naoki Aizu, Takumi Kito, Kazuhiro Nishii, Kouji Yamada

**Affiliations:** 1 Faculty of Rehabilitation, School of Health Sciences, Fujita Health University, Toyoake, Aichi, Japan; 2 Graduate School of Health Sciences, Fujita Health University, Toyoake, Aichi, Japan; 3 Department of Physical Therapy, Faculty of Health Sciences, Kinjo University, Hakusan, Ishikawa, Japan; Brunel University London, UNITED KINGDOM OF GREAT BRITAIN AND NORTHERN IRELAND

## Abstract

This study aimed to elucidate the effects of the tibialis anterior (TA) muscle contraction on the medial longitudinal arch (MLA) and hallux valgus (HV) angle, primarily focusing on the potential functional role of the TA in foot alignment. Twenty-five healthy adults (mean age: 20.7 ± 1.0 years) participated. Electrical stimulation was applied to the TA without ankle dorsiflexion. A triaxial accelerometer was attached to the skin over the navicular bone, and electrogoniometers were placed over the interphalangeal joints of the hallux and ankle. The navicular displacement was calculated through double integration of the acceleration data. Contraction of the TA caused navicular displacement in the supination direction (upward, posterior, and medial), suggesting a temporary elevation of the MLA. The hallux showed an average varus shift of 1.36°; the shift was observed in 24 of 25 participants (p < 0.001). A significant positive correlation was found between upward displacement of the navicular bone and the extent of varus change in the HV angle (r = 0.463, p = 0.020). Additionally, a greater initial HV angle was associated with a larger varus shift in the hallux after TA contraction (r = 0.433, p = 0.030). No significant correlations were observed between the HV angle change and ankle dorsiflexion, suggesting that the medial hallux shift may result from structural changes in the MLA rather than isolated joint motion. These findings provide novel evidence that TA contractions can elevate the MLA and induce varus displacement of the hallux. These findings further suggest that the TA contributes to foot biomechanics not only as an isolated muscle but also as part of a coordinated muscle system involved in arch dynamics and toe alignment. This suggests the potential clinical applications of TA activation in noninvasive interventions for mild-to-moderate HV, such as exercise therapy or neuromuscular electrical stimulation.

## Introduction

The tibialis anterior (TA) muscle contributes to the dorsiflexion and supination of the ankle joint and plays a key role in controlling foot motion [1,2]. Its insertion into the medial cuneiform and first metatarsal bones is anatomically close to the medial longitudinal arch (MLA) [1,3]. However, there is limited evidence on whether TA directly support MLA. Traditionally, the tibialis posterior and intrinsic foot muscles have been considered the primary contributors to MLA stability [4,5]. In contrast, in patients with pes planus, which is characterized by a lowered arch height, compensatory activation of the TA muscle has been reported due to the dysfunction of intrinsic muscles such as the flexor digitorum brevis [6,7]. These findings suggest that the TA, which induces foot supination, may play a compensatory role in arch maintenance under certain conditions; however, this role remains unclear.

Recent biomechanical studies have proposed that the foot should not be interpreted as a collection of isolated muscles but rather as an integrated system of muscle groups that collectively determine the structure of the foot arches and toe alignment [8]. In particular, the tibialis anterior and fibularis longus muscles form a functional “tendon stirrup,” which contributes to stabilization and elevation of the medial longitudinal arch through coordinated action [9]. This perspective suggests that the tibialis anterior may contribute to arch support not merely through its anatomical proximity, but as part of a dynamic muscle system. However, experimental evidence demonstrating this functional contribution remains limited.

The MLA comprises the calcaneus, talus, navicular, medial cuneiform, and first metatarsal; it plays a crucial role in shock absorption and load distribution during weight-bearing [3,10]. In addition to skeletal structures, active support from muscles such as the tibialis posterior, flexor digitorum longus, and flexor hallucis longus is essential for stability of the MLA [4]. A decrease in the MLA height, as observed in flatfoot, promotes excessive foot pronation and increases valgus stress on the forefoot, thereby contributing to the development of hallux valgus (HV) [11,12]. HV is highly prevalent in the elderly population, with reports indicating a prevalence exceeding 40% among women aged ≥65 years [13]. Moderate to high correlations have been reported between HV severity and decreased arch height [13–15]. These findings strongly suggest that deterioration of medial arch function is likely to contribute to the progression of HV.

This study aimed to clarify the effects of TA muscle contraction on the MLA and HV angles. In particular, we aimed to investigate whether isolated contraction of the TA could lead to elevation of the MLA, and consequently, to changes in the HV angle. Despite its anatomical proximity to bones comprising the MLA, the functional contribution of the TA has not been adequately explored. Therefore, we adopted a non-invasive approach to assess the effects of direct TA contraction. We hypothesized that contraction of the TA would elevate the MLA and improve the HV angle by inducing a medial deviation of the hallux. Elucidation of the novel functional role of TA could contribute to the development of new exercise-based therapies and preventive strategies for HV.

## Methods

### Participants

Twenty-five healthy adults (11 male; mean age: 20.7 ± 1.0 years; height: 164.6 ± 10.1 cm; weight: 57.2 ± 10.4 kg) participated in this study. Participants were recruited through recruitment posters at the university. The HV angle was defined as the angle between the longitudinal axes of the first metatarsal and the proximal phalanx of the hallux [16]. The anatomical landmarks of the first metatarsal and proximal phalanx were identified via palpation, and all measurements were conducted manually by the same examiner using a goniometer placed on the skin after sufficient practice with the measurement procedure [17,18]. Fourteen participants had normal HV angles, ten had mild HV (20–30°), and one had moderate HV (30–40°). Foot dominance was determined via a standardized questionnaire [19]; 22 and 3 of the participants were right-footed and left-footed, respectively (mean score: 72.7 ± 48.3).

Participants were prospectively recruited between May 18, 2022, and March 31, 2025. The study procedures were explained orally using a written document, and written informed consent was obtained by participants signing the consent form. This study was approved by the Fujita Health University Ethics Committee (Approval No.: HM21–367). A post hoc sample size estimation was performed using G*Power version 3.1.9.7 for a paired-samples t-test based on the observed effect size for the change in hallux valgus angle induced by tibialis anterior contraction. The present sample size was considered sufficient with an alpha level of 0.05 and a statistical power of 0.80.

### Experimental methods

**Fig 1:**
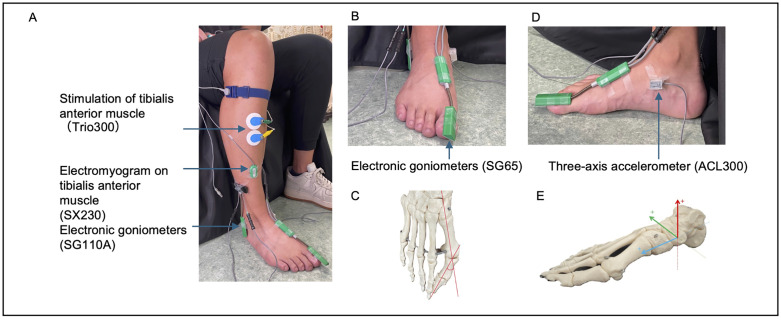
Experimental setup. A: The tibialis anterior muscle contracting through electrical stimulation. To measure muscle contraction and ankle joint movement, an electromyogram was applied to the skin just above the tibialis anterior muscle and an electrogoniometer was applied to the ankle and hallux metatarsophalangeal joints. B, C: To measure the hallux valgus angle, an electronic goniometer is attached to the hallux metatarsophalangeal joint. The hallux valgus angle was measured as the angle between the first metatarsal bone and first basal phalanx. D, E: Acceleration of the navicular bone caused by contraction of the tibialis anterior muscle was estimated, and the movement of the navicular bone was calculated. All images are original photographs taken by the authors, and all annotations were added by the authors.

Selective electrical stimulation of the TA was performed to examine its effects on the elevation of MLA and HV angles. A Trio300 stimulator (ITO Co., Ltd., Tokyo, Japan) was used (Fig 1). Stimulation parameters were carefully set to elicit minimal TA contraction—sufficient to activate the muscle but without producing observable dorsiflexion—using 0.5 seconds of stimulation followed by 1.0 second of rest, repeated 10 times, to prevent dorsiflexion that might affect the MLA and HV angle in the measurement (see below). During testing, the participants were seated with the hip and knee joints flexed at 90° and the ankle in a neutral position (0°). They were instructed to remain relaxed and avoid voluntary muscle activation during stimulation.

### Measurement devices and variables

Changes in HV angle, ankle dorsiflexion, and hallux metatarsophalangeal (MTP) joint motion due to TA contraction were measured using skin-mounted electrogoniometers (Biometrics Ltd., Newport, UK) (Fig 1). The reliability of this device has been previously validated in prior study [20]. Specifically, the HV angle (positive, valgus; negative, varus) and the hallux MTP flexion/extension (positive, extension; negative, flexion) were measured using SG65 sensors attached to the skin over the first metatarsal and proximal phalanx. Ankle dorsiflexion (positive: dorsiflexion; negative: plantar flexion) was measured using an SG110A sensor. To evaluate navicular bone motion as an indicator of MLA dynamics [21,22], a triaxial accelerometer (ACL300; Biometrics Ltd., Newport, UK) was attached to the skin over the navicular bone. Acceleration data in all three axes were collected during TA contraction and double-integrated to calculate displacement. Acceleration values recorded in gravitational units (g) were converted to m/s² prior to integration. All data were digitized using PowerLab (ADInstruments Pty Ltd., Sydney, NSW, Australia) and recorded and analyzed using LabChart software (ADInstruments Pty Ltd., Sydney, NSW, Australia).

### Statistical analysis

All statistical analyses were performed using SPSS version 26 (IBM Corp., Armonk, NY, USA). Data normality was assessed using the Shapiro–Wilk test. Pearson’s correlation coefficients were calculated for normally distributed variables, whereas Spearman’s rank correlation coefficients were used for non-normally distributed variables. Exact binomial tests were used to evaluate the significance of directional changes in navicular displacement and hallux valgus angle changes before and after stimulation. Statistical significance was set at p < 0.05.

## Results

### Displacement of the navicular bone

Following electrical stimulation of the TA, the displacement of the navicular bone was confirmed by calculating the displacement from the triaxial accelerometer data using double integration (Table 1).

**Table 1:** Directional displacement of the navicular bone induced by the tibialis anterior muscle contraction.

Plane of Motion	Direction	Number of Participants	Total	*p*-value (Exact binomial test)
**Superior–Inferior**	**Superior**	**25**	**25**	***p* < 0.001**
	**Inferior**	**0**		
**Anterior–Posterior**	**Anterior**	**4**	**25**	***p* = 0.001**
	**Posterior**	**21**		
**Medial–Lateral**	**Medial**	**23**	**25**	***p* < 0.001**
	**Lateral**	**2**		

Specifically, contraction of the TA was found to induce navicular movement in the superior, posterior, and medial directions, indicating displacement in the direction of foot supination. Although ankle dorsiflexion was not visually observed, the mean change in the ankle dorsiflexion angle measured using an electrogoniometer was 0.21°. To confirm that the navicular displacement was not due to ankle dorsiflexion, we examined the correlation between the change in ankle dorsiflexion angle and superior acceleration of the navicular bone. No significant correlation was observed (Spearman r = 0.309, p = 0.139).

### Changes in the HV angle

**Fig 2:**
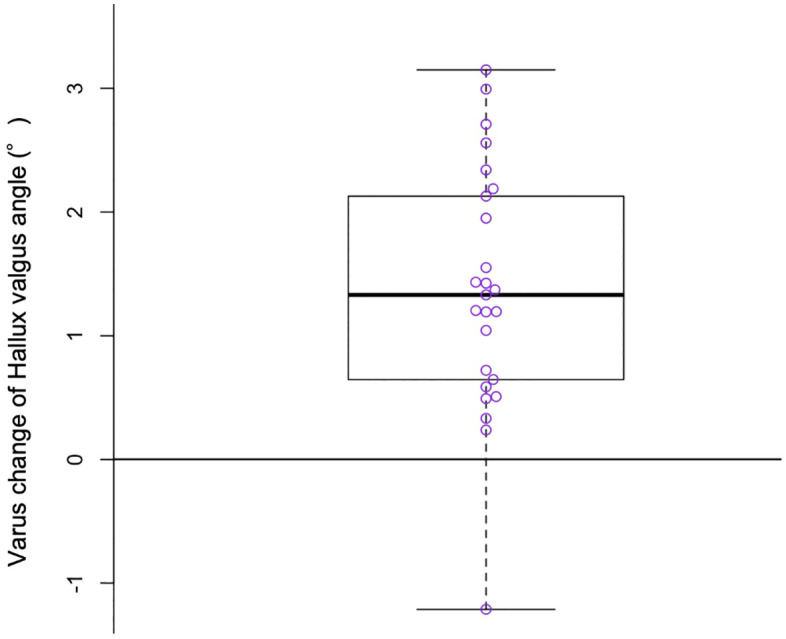
The contraction of the tibialis anterior muscle caused the hallux valgus angle to move varus. A varus change was observed in 24 of the 25 participants.

The TA contraction resulted in an average varus shift of 1.36° in the HV angle. Varus changes were observed in 24 of the 25 participants. Exact binomial testing revealed that this change was statistically significant (p < 0.001, Fig 2).

### Relationship between navicular displacement and HV angle

**Fig 3:**
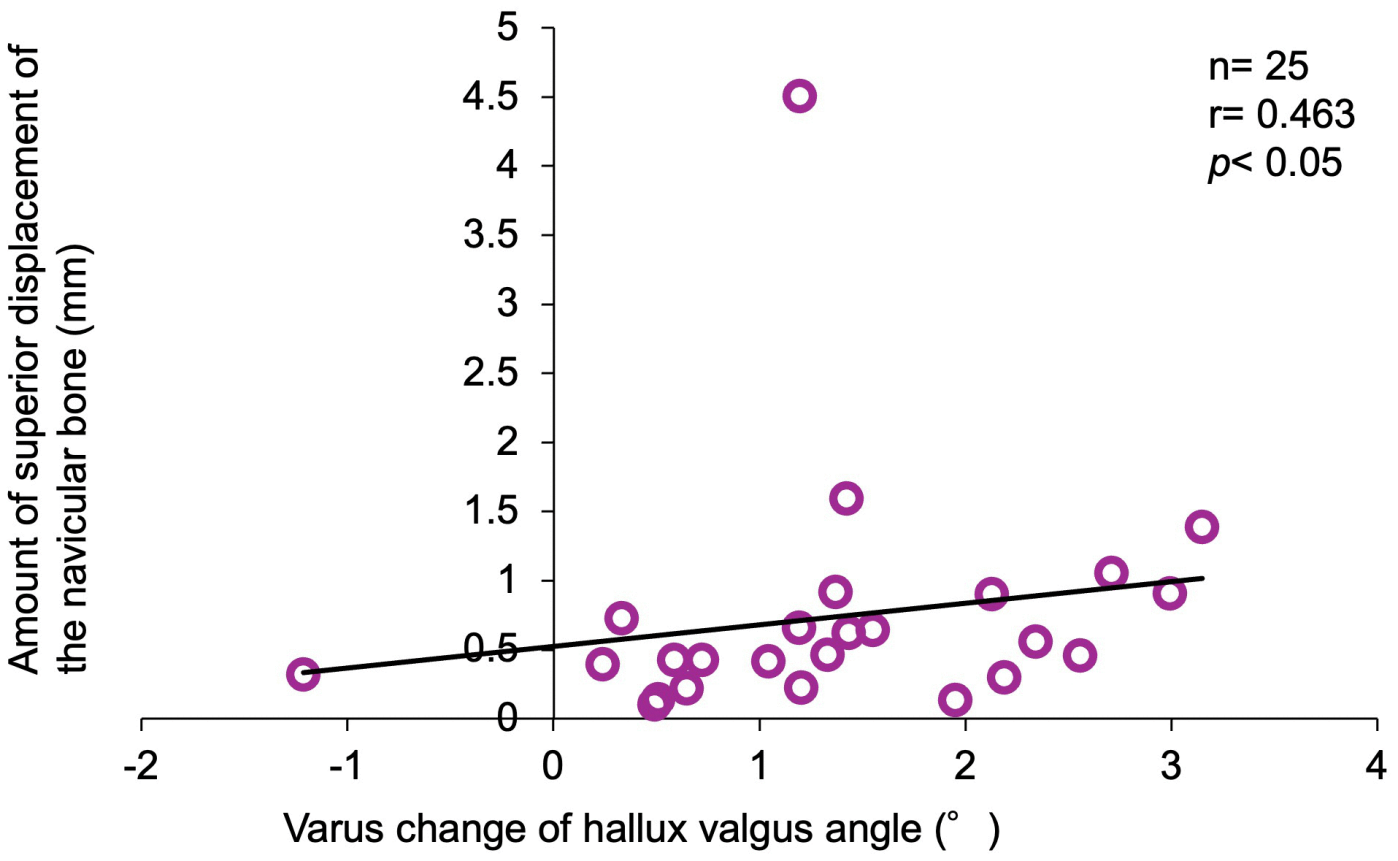
The superior displacement of the navicular was positively correlated with varus change of hallux valgus angle. A greater upward displacement of the navicular region was associated with a greater medial shift of the hallux.

A significant positive correlation was observed between superior displacement of the navicular bone and the extent of change in the HV angle (Spearman’s r = 0.463, p = 0.020, Fig 3). This indicates that a greater upward displacement of the navicular bone was associated with a greater medial shift of the hallux. To examine whether the HV angle change was influenced by other factors, we examined the correlations between the HV angle change and changes in the ankle dorsiflexion/plantarflexion angle and hallux MTP flexion/extension angle. No significant correlations were found in either case (ankle: r = 0.312, p = 0.129; hallux: r = 0.134, p = 0.524).

### Relationship between initial HV angle and its change

**Fig 4:**
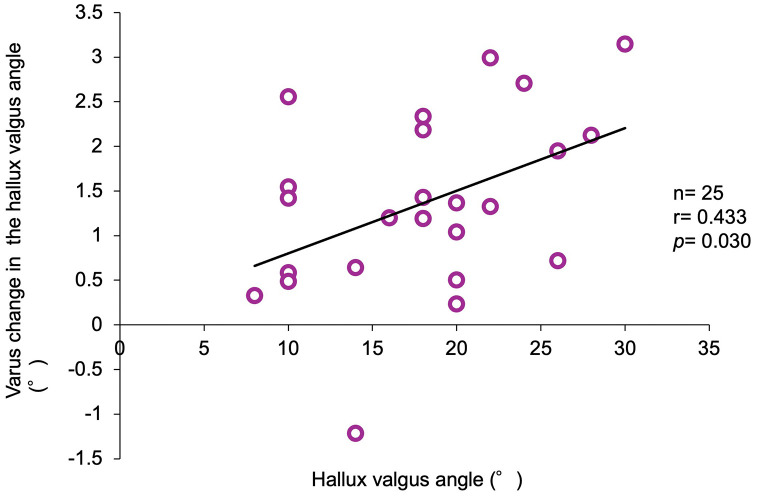
The positive correlation between the initial hallux valgus angle and the extent of varus change in the hallux valgus angle elicited by contraction of tibialis anterior muscle. The greater the hallux valgus angle, the greater is the extent of varus change in the hallux valgus angle.

A significant positive correlation was found between the initial HV angle at rest and the extent of varus shift induced by TA contraction (Pearson’s r = 0.433, p = 0.030; Fig 4). This suggests that the participants with larger initial HV angles tended to show greater medial correction of the hallux after TA contraction.

## Discussion

This study aimed to clarify the effects of TA contraction on the MLA and HV angles. We applied electrical stimulation to the TA in healthy adults and measured the navicular acceleration, HV angle, and joint angles of the ankle and hallux using a noninvasive method. The TA contraction was found to induce navicular displacement in the superior, posterior, and medial directions, corresponding to foot supination. Additionally, the HV angle changed by an average of 1.36° in the medial direction in 24 of 25 participants. A significant positive correlation was found between the extent of the superior displacement of the navicular bone and changes in the HV angle. In contrast, HV angle changes were not significantly correlated with ankle dorsiflexion or hallux flexion-extension angles. Furthermore, a larger initial HV angle was associated with a larger medial shift, suggesting that TA contraction contributes directly to MLA support and may influence the HV angle through navicular elevation, thereby offering a new anatomical and functional perspective.

The posterior tibialis muscle is widely recognized as the primary dynamic supporter of the MLA, and several studies have confirmed its arch-supporting function through its attachment to the navicular bone [4,23]. However, the role of the TA in MLA support remains unclear. In the present study, the TA contraction clearly induced navicular displacement in the supination direction (superior, posterior, and medial), suggesting its involvement in temporary arch elevation. This finding is supported by motion analysis of the talonavicular joint, which demonstrated the coupling of arch elevation and foot supination [24], and recent dynamic imaging study that have confirmed this relationship [25]. Additionally, intrinsic foot muscles, such as the flexor digitorum brevis and abductor hallucis, contribute to MLA support [26,27]. The TA may compensate for arch support in patients with flat feet and impaired intrinsic muscle functions [6]. These findings can be further interpreted within the framework of recent biomechanical models of foot function that emphasize coordinated muscle systems in foot function [8]. In particular, the tibialis anterior and fibularis longus muscles are thought to form a functional “tendon stirrup,” which contributes to stabilization and elevation of the medial longitudinal arch through their combined action [9]. Although this tendon stirrup concept has been proposed based on anatomical and theoretical considerations, direct experimental evidence has been limited. The present findings provide experimental support for this model by demonstrating that isolated activation of the tibialis anterior can induce navicular elevation and medial shift of the hallux. This suggests that the tibialis anterior plays a more integral role in foot arch dynamics and toe alignment than previously recognized, functioning not merely as an accessory muscle but as part of a coordinated muscle system.

The TA contraction produced displacement of the navicular bone toward supination and a mean medial shift of 1.36° in the hallux. This suggests that the TA activity affects the medial foot structures and tension balance, generating a varus moment on the hallux. HV is characterized by medial deviation of the first metatarsal and lateral deviation of the hallux and is associated with MLA collapse and excessive pronation [28]. The observed navicular supination may enhance medial structural support and suppress the valgus moment. The observed coordination between supination and the medial hallux shift aligns with previous findings that link arch elevation with foot supination [24] and dynamic imaging results that report simultaneous arch elevation and supination [25]. A significant positive correlation was also found between superior displacement of the navicular bone and medial shift of the hallux. In contrast, the HV angle changes were not significantly correlated with ankle dorsiflexion (r = 0.312, p = 0.129) or hallux MTP motion (r = 0.134, p = 0.524), suggesting that the medial hallux shift reflects structural changes in the MLA rather than isolated joint movements. These findings highlight the influence of MLA dynamics on HV angle and underscore the importance of deep support structures in maintaining hallux alignment.

A significant positive correlation was observed between the initial HV angle at rest and magnitude of medial correction following TA contraction. This suggests that more severe HV deformities may be more responsive to TA-induced correction, reflecting structural and mechanical responsiveness. As HV progresses, ligamentous laxity, joint capsule attenuation, bony misalignment, and reduced intrinsic muscle activity may influence its reversibility [29]. TA contraction may exert greater corrective effects in early stage deformities where dynamic muscle control is more effective. However, this study mostly included participants with normal or mild HV; only one subject had moderate HV and none had severe deformities. Thus, the findings are limited to early stage HV, and generalizations of these findings to more advanced cases should be made with caution. These results suggest that dynamic responsiveness, stratified according to HV severity, can aid in evaluating the timing and efficacy of conservative interventions.

Our results indicate that TA contraction induces displacement of the navicular bone in the direction of supination, elevates the MLA, and medially shifts the hallux. These findings suggest that TA activity may help correct foot alignment, which has therapeutic potential for the management of mild-to-moderate HV. Functional activation of the TA may be incorporated into rehabilitation strategies such as exercise therapy or neuromuscular electrical stimulation (NMES), serving as a noninvasive alternative to orthotic devices or surgery. Orthoses have been shown to be effective in improving pain and alignment in mild-to-moderate HV [30], whereas surgery is generally reserved for severe deformities [31].

Although these findings provide novel insights into the biomechanical effects of TA contractions, several considerations should be noted. The present study employed a short-term single-stimulation protocol in healthy or mildly deformed individuals, which did not reflect the effects of long-term intervention or sustained use. Navicular displacement was estimated using skin-mounted accelerometers that are potentially subject to skin motion artifacts [32–34]. Furthermore, given the multifactorial nature of HV progression, the mechanical responses observed in this study may not be generalizable to other populations. Future studies incorporating more severe cases, broader age groups, and longitudinal data with advanced motion analyses are warranted. Despite these limitations, the present findings provide important insights into the biomechanical role of the tibialis anterior. These findings may help bridge the gap between anatomical theory and functional evidence in foot biomechanics, particularly by clarifying the role of the tibialis anterior within a coordinated muscle system.

## Conclusion

The TA contraction displaced the navicular bone in the direction of supination, elevated the MLA, and induced medial shifting of the hallux. These findings suggest that activity of the TA contributes to foot alignment and may serve as a valuable conservative intervention for mild to moderate HV. Importantly, these findings indicate that the tibialis anterior plays a functional role in foot biomechanics not only as an isolated muscle but also as part of a coordinated muscle system contributing to arch stability and toe alignment. Future application of this approach to patients with HV deformities, rather than healthy individuals, may provide clearer insights and contribute to the development of more effective therapeutic strategies.
